# The rule of fives, a simple way to stratify risk for primary gastrointestinal stromal tumors (GIST)

**DOI:** 10.1186/2045-3329-2-21

**Published:** 2012-10-05

**Authors:** Robert G Maki

**Affiliations:** 1 Departments of Medicine, Pediatrics, and Orthopaedics, Mount Sinai School of Medicine, New York, NY, 10029-6574, USA

**Keywords:** Gastrointestinal stromal tumor, GIST, Staging, Adjuvant therapy

## Abstract

The current AJCC version 7 staging system is a lugubrious means to classify gastrointestinal stromal tumors with respect to their risk and need for adjuvant systemic therapy. The rule of fives, suggested here, is a means to quickly assess whether a given gastrointestinal stromal tumor is low vs intermediate-high risk.

## Correspondence

Risk stratification for primary gastrointestinal stromal tumors (GISTs) has evolved with the understanding of the disease, spurred by its demonstration as a specific sarcoma subtype. GISTs most commonly contain an activating mutation in *KIT*, or much less commonly in *PDGFRA*; even rarer forms of GIST have other genetic alterations. Risk stratification is critical, since three years of adjuvant imatinib is now considered a standard of care for patients with intermediate to high risk primary GIST.

Risk criteria were first published in 2001 and were amended as more was learned about the importance of mitotic rate, anatomic primary site, and size, in large part due to large case series developed by Miettinen, Lasota and colleagues [1,2]. These factors have been incorporated into a nomogram [3], risk heat map and detailed staging system [4], and have been incorporated into the most recent (7^th^) edition of the American Joint Committee on Cancer (AJCC) staging manual.

The nomenclature for staging involves a number of details that may be difficult to remember for an uncommon tumor. The collective data suggest a simple way to deduce low risk vs. intermediate-high risk GISTs. The “rule of fives” states that intermediate to high risk *gastric* GISTs are *both* more than 5 cm in size *and* have more than 5 mitoses (mit) per 50 high powered fields (hpf). Conversely, *nonCXgastric* GISTs are high grade if they are *either* more than 5 cm in size *or* have more than 5 mit/50 hpf. With this simple “and/or” construct, all the GISTs with over 50% risk of recurrence are captured, as are an intermediate risk group of 5–10 cm small bowel GIST with low mitotic rate (24% recurrence risk, Miettinen prognostic group 3a).

This rule is particularly useful to highlight the situation of a common but surprisingly low risk GIST, i.e. those gastric GISTs that are >5 cm and have a low mitotic rate. Since the recurrence risk of these GISTs is 12% or less per Miettinen and colleagues [1,2], these patients are potentially good candidates for observation alone after primary surgery.

Further nuances will arise as exact mutation rate and *KIT* or *PDGFRA* mutation status are incorporated into staging. However, exon 9 *KIT* mutations (~15% of GIST) are nearly exclusively found in a small bowel primary site, and uncommon *PDGFRA* mutations are usually associated with a gastric primary site. As a result, future patient staging efforts should not prove unduly cumbersome, and can build on this simple rule.

## Author’s contributions

The author was solely responsible for the content of this manuscript, including literature search, data interpretation, and writing.

## Competing interest

Dr Maki has received speaking fees from Novartis.

## Role of the funding source

There is no relevant research support to report.
